# Resting state EEG theta/beta power ratio and cognitive reserve in cognitively unimpaired older adults

**DOI:** 10.1002/alz.095513

**Published:** 2025-01-09

**Authors:** Laura E. Korthauer, Julia Sinople, Zachary T Gemelli, William C Heindel, Elena K Festa

**Affiliations:** ^1^ Rhode Island Hospital, Providence, RI USA; ^2^ Alpert Medical School Brown University, Providence, RI USA; ^3^ University of Wisconsin‐Milwaukee, Milwaukee, WI USA; ^4^ Brown University, Providence, RI USA

## Abstract

**Background:**

Patients with mild cognitive impairment (MCI) and dementia due to Alzheimer’s disease show a pattern of “slowing” on resting state electroencephalography (EEG), often indexed by ratio of theta to beta power. The objective of this study was to investigate associations between theta/beta power ratio, subjective cognitive decline, and cognitive reserve in cognitively unimpaired older adults at high versus low risk for AD.

**Method:**

Cognitively unimpaired older adults at high risk (*APOE e*4 carrier and positive family history; N = 25) or low risk (*APOE* non‐*e*4 carrier and negative family history; N = 25) for AD completed questionnaires about subjective cognitive decline (SCD; Everyday Cognition Scale) and cognitive reserve (Cognitive Reserve Index Questionnaire). Five minutes of eyes‐open 64‐channel resting state EEG was acquired. Data were separated into 2s epochs for analysis, and ratio of theta to beta power at left (F3, F5, F7), mid (Fz, FCz, Cz), and right (F4, F6, F8) frontal electrodes was calculated.

**Result:**

After controlling for age, there was a significant positive association between cognitive reserve index and theta/beta power across left, right, and midfrontal regions (all *r*’s > .30, *p*’s < .03). The magnitude of these associations did not differ between people at high versus low risk for AD. However, only the high‐risk group showed a significant positive association between higher right and left frontal theta/beta ratio and greater SCD symptoms (*r*’s > .4, *p*’s < .05).

**Conclusion:**

A pattern of EEG slowing (higher theta/beta ratio) was associated with greater SCD selectively in those at high risk for AD. Interestingly, increased EEG slowing was also associated with greater cognitive reserve in cognitively unimpaired older adults. One potential explanation for these findings is that people with higher cognitive reserve are able to maintain a cognitively unimpaired state despite the presence of AD‐related EEG slowing while those with lower cognitive reserve cannot; however, biomarker studies are needed to clarify.

